# Global and regional estimates of tuberculosis burden attributed to high fasting plasma glucose from 1990 to 2019: emphasis on earlier glycemic control

**DOI:** 10.1186/s12889-024-18260-z

**Published:** 2024-03-13

**Authors:** Qin Bian, Yanjun Zhang, Chen Xue, Wenjing Lu, Wei Li, Fanqi Pan, Yi Li

**Affiliations:** Department of Disease Control and Prevention, Shanghai Changhai Hospital, Shanghai, China

**Keywords:** Global burden, Tuberculosis, High fasting plasma glucose, Socio-demographic indexes, Glycemic control

## Abstract

**Background:**

Previous studies have shown subjects suffering from diabetes or persistent hyperglycemia were more likely to develop tuberculosis (TB). However, the global burden of TB attributed to high fasting plasma glucose (HFPG) remains unclear. This study aimed to characterize the global, regional, and national TB burden attributed to HFPG from 1990 to 2019.

**Methods:**

With Global Burden of Disease study 2019, the numbers and age-standardized mortality rates (ASMR) and age-standardized disability-adjusted life years (DALY) rates (ASDR) of TB attributed to HFPG at global, regional, and national levels from 1990 to 2019 were extracted. The locally weighted regression model was applied to estimate the TB burden for different socio-demographic index (SDI) regions.

**Results:**

Globally, the ASMR and ASDR attributed to HFPG were 2.70 (95% UI, 1.64–3.94) and 79.70 (95% UI, 50.26–112.51) per 100,000 population in 1990, respectively. These rates decreased to 1.46 (95% UI, 0.91–2.08) and 45.53 (95% UI, 29.06–62.29) in 2019. The TB burden attributed to HFPG remained high in low SDI and Central Sub-Saharan Africa regions, while it declined with most significantly in high SDI and East Asia regions. Additionally, the ASMR and ASDR of TB attributed to HFPG were significantly higher in the male and the elderly population.

**Conclusions:**

The global TB burden attributable to HFPG decreased from 1990 to 2019, but remained high in low SDI regions among high-risk populations. Thus, urgent efforts are required to enhance the awareness of early glycemic control and TB treatment to alleviate the severe situation.

**Supplementary Information:**

The online version contains supplementary material available at 10.1186/s12889-024-18260-z.

## Introduction

High fasting plasma glucose (HFPG), defined as fasting plasma glucose above 86.4–97.2 mg/dl, was globally responsible for 11.3% of all age-standardized deaths and 6.4% of the disability-adjusted life-years (DALYs) from all causes. Previous studies have shown that subjects suffering from diabetes or persistent hyperglycemia are more likely to be infected with several infectious diseases [1] and experience poor therapeutic effects [2]. Among these infectious diseases, tuberculosis (TB) is one of them extensively affected by HFPG.

Diabetes or hyperglycemia has been considered as the primary challenge among the risk factors hindering TB control by 2035 [3]. Diabetes has been reported to be associated with genotypically drug-resistant TB [4]. Another case-control study revealed that TB-diabetes patients were associated with increased levels of circulating angiogenic factors, indicating that angiogenesis disorders and excessive inflammation may be involved in the occurrence of TB-diabetes [5]. An epidemiological study in China demonstrated that patients with diabetes present a higher risk of TB treatment failure [6]. Additionally, a prospective study conducted in Brazil suggested that diabetes result in an increased risk of both TB mortality and TB treatment failure [2]. Although the relationship between diabetes or hyperglycemia and TB has been gradually clarified, there remains a lack of systematic research on the burden of TB attributed to HFPG on a global scale.

In this study, we aimed to reported the trend of HFPG-related TB burden by age, sex, sociodemographic index (SDI) between 1990 and 2019 using data from the GBD 2019 study. These findings could be helpful for government resource allocation by government, the implementation of screening programs for high-risk populations, and disseminating knowledge of early glycemic control among people.

## Methods

### Data sources

The Global Burden of Disease (GBD) project, which is conducted by the Institute for Health Metrics and Evaluation (IHME), explores the levels and trends of communicable diseases, non-communicable diseases and injuries worldwide [7]. The Global Health Data Exchange (GHDx, http://ghdx.healthdata.org/gbd-results-tool) was adopted to extract the data. The data included age-standardized mortality rate (ASMR) per 100k population and age-standardized disability-adjusted life rate (ASDR) per 100 k population for TB attributable to HFPG, as well as socio-demographic index (SDI), covering 204 countries and regions around the world from 1990 to 2019. Disability-adjusted life years (DALYs) were obtained with the sum of the number of years of life lost due to premature death (YLLs) and the number of years of life lost due to disability (YLDs).

### Definition of the TB, HFPG and SDI

In GBD 2019, TB cases were identified based on the International Classification of Diseases, version 10 (ICD-10). The discharge diagnosis codes for HIV-negative tuberculosis are A10–19.9, B90–90.9, K67.3, K93.0, M49.0, and P37.0; for HIV-positive tuberculosis, the ICD 10 code is B20.0 [8]. Fasting plasma glucose (FPG) was measured as the mean FPG in a population, where FPG is a continuous exposure in units of mmol/L. Due to the fact that FPG is a continuous variable, IHME defines high FPG as any level above the theoretical minimum risk exposure level (TMREL), which is 4.8–5.4 mmol/L or 86.4–97.2 mg/dL [9]. Social Development Index (SDI) is an important metric used to comprehensively evaluate the level of social development for each country or region [10]. In GBD 2019, countries and regions were divided into five levels based on SDI: high SDI (greater than 0.81), high-middle SDI (0.70–0.81), middle SDI (0.61–0.69), low-middle SDI (0.46–0.60), and low SDI (less than 0.46). SDI allows for comparisons among different geographies and regions and has been explored in numerous studies [11, 12].

### Statistical analysis

Differences in age structure may result in the heterogeneity of TB burden quantified by mortality and DALYs. To eliminate the influence of age structure differences, we used the age-standardized mortality rate (ASMR) and age-standardized disability-adjusted life rate (ASDR) were utilized to estimate TB burden. Percentage change, annual percentage change (APCs), and average annual percentage change (AAPCs) were adopted to imply the trend in TB ASMR or ASDR attributable to HFPG from 1990 to 2019. Furthermore, the Pearson correlation was conducted and the Pearson correlation coefficient was used to show the correlation between SDI with ASMR and ASDR, as well as obtained the expected relationship between SDI with ASMR and ASDR by the Gaussian process regression model fitting of data obtained from 1990 to 2019. The Gaussian process regression model was established by the “kernlab” package [13] of R software (version 4.1.0).

## Results

### Global tuberculosis (TB) burden attributable to high fasting plasma glucose (HFPG) from 1990 to 2019

**Fig. 1:**
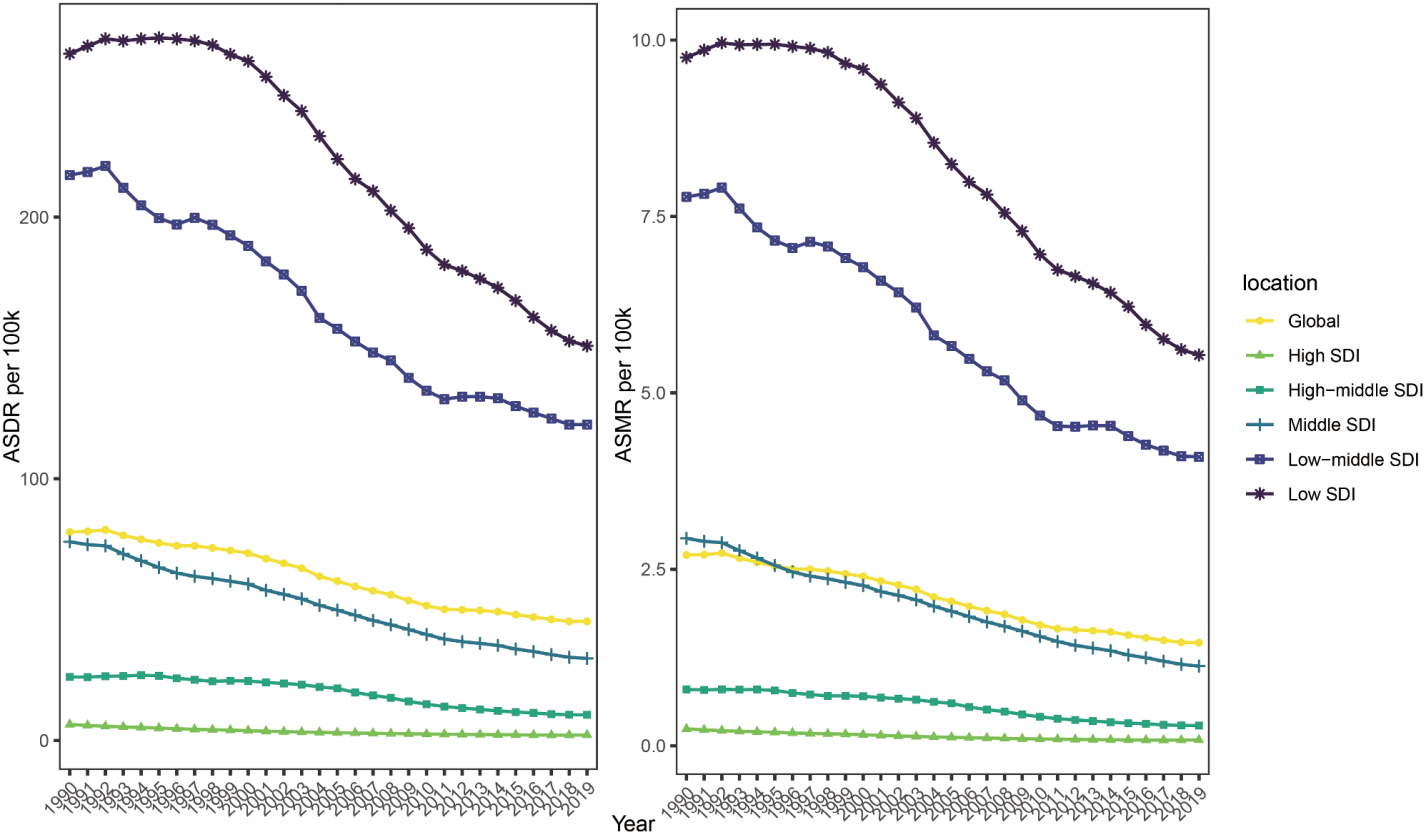
The ASMR and ASDR of tuberculosis attributable to HFPG from 1990 to 2019. ASMR, Age-standardized mortality rates; ASDR, Age-standardized disability-adjusted life years rates; SDI, socio-demographic index; HFPG, high fasting plasma glucose

In the global analysis of different countries and regions, we described the trends in TB ASMR and ASDR attributable to HFPG from 1990 to 2019 (Fig. 1). Notably, the TB burden attributable to HFPG remained high levels in low SDI and low-middle SDI regions, both of which indicated decreasing trends from 1990 to 2019. Globally, the TB ASMR and ASDR attributable to HFPG were 2.70 and 79.70 in 1990, while were 1.46 and 45.53 respectively in 2019.

The TB ASMR and ASDR attributable to HFPG were influenced by genders, SDI levels and different GBD regions (Tables 1 and 2). In 1990 and 2019, the TB ASMR and ASDR attributable to HFPG in male were higher than in female. The trends of TB ASMR and ASDR attributable to HFPG in different SDI regions were all decreasing. The region with the largest decline in ASMR and ASDR was the high SDI region (with an Estimated Annual Percent Change (EAPC) of −65.68% and − 65.54%), while the region with the lowest decline was the low SDI region (with an EAPC of −0.43% and − 42.54%). The trends of TB ASMR and ASDR attributable to HFPG in most GBD regions are decreasing, especially in East Asia (EAPC were − 86.75% and − 84.07%), Andean Latin America (EAPC were − 76.66% and − 76.87%) and High-income Asia Pacific (EAPC were − 76.19% and − 80.66%). However, these rates were increasing in Southern Sub-Saharan Africa (EAPC were 14.23% and 10.48%), Central Asia (EAPC were 11.36% and 10.80%) and Eastern Europe (EAPC were 9.61% and 19.09%) (Tables 1 and 2).

**Table 1:** The ASMR and EAPC in TB attributed to high fasting plasma glucose from 1990 to 2019

Group	ASMR,1990	ASMR,2019	EAPC
Global	2.70(1.64,3.94)	1.46(0.91,2.08)	−45.99%
**Sex**			
Male	3.93(2.37,5.76)	2.10(1.28,3.01)	−46.69%
Female	1.70(1.00,2.53)	0.89(0.53,1.33)	−47.41%
**SDI levels**			
High SDI	0.24(0.14,0.36)	0.08(0.05,0.12)	−65.68%
High-middle SDI	0.80(0.49,1.16)	0.29(0.18,0.41)	−64.16%
Middle SDI	2.94(1.66,4.47)	1.13(0.66,1.69)	−61.62%
Low-middle SDI	7.78(4.64,11.63)	4.09(2.47,6.00)	−47.37%
Low SDI	9.75(5.70,14.71)	5.54(3.21,8.21)	−43.18%
**GDB regions**			
Andean Latin America	2.79(1.60,4.32)	0.65(0.36,1.02)	−76.66%
Australasia	0.04(0.02,0.06)	0.02(0.01,0.04)	−44.72%
Caribbean	0.82(0.51,1.22)	0.50(0.32,0.72)	−39.17%
Central Asia	0.57(0.35,0.79)	0.64(0.40,0.90)	11.36%
Central Europe	0.38(0.23,0.55)	0.14(0.09,0.20)	−62.64%
Central Latin America	1.72(1.02,2.54)	0.38(0.23,0.55)	−77.81%
Central Sub-Saharan Africa	13.96(7.78,22.15)	10.84(6.05,17.04)	−22.37%
East Asia	1.52(0.85,2.32)	0.20(0.11,0.31)	−86.75%
Eastern Europe	0.27(0.17,0.39)	0.30(0.18,0.43)	9.61%
Eastern Sub-Saharan Africa	12.79(7.42,19.25)	6.29(3.67,9.51)	−50.85%
High-income Asia Pacific	0.56(0.31,0.85)	0.13(0.07,0.22)	−76.19%
High-income North America	0.08(0.04,0.11)	0.03(0.02,0.04)	−62.01%
North Africa and Middle East	1.18(0.65,1.85)	0.49(0.28,0.73)	−58.70%
Oceania	4.92(2.72,7.83)	3.75(2.09,5.82)	−23.85%
South Asia	9.56(5.70,14.33)	4.64(2.80,6.85)	−51.43%
Southeast Asia	5.98(3.24,9.50)	2.75(1.51,4.20)	−54.01%
Southern Latin America	0.40(0.23,0.61)	0.18(0.10,0.26)	−55.58%
Southern Sub-Saharan Africa	5.02(2.98,7.44)	5.74(3.44,8.27)	14.23%
Tropical Latin America	0.79(0.49,1.14)	0.26(0.16,0.37)	−67.58%
Western Europe	0.14(0.07,0.21)	0.04(0.02,0.07)	−68.17%
Western Sub-Saharan Africa	6.19(3.22,9.91)	4.10(2.10,6.60)	−33.73%

ASMR, Age-standardized mortality rates; EAPC, Estimated annual percent change; SDI, socio-demographic index

**Table 2:** The ASDR and EAPC in TB attributed to high fasting plasma glucose from 1990 to 2019

Group	ASDR,1990	ASDR,2019	EAPC
Global	79.70(50.26,112.51)	45.53(29.06,62.29)	−42.88%
**Sex**			
Male	111.03(71.27,158.02)	64.57(41.18,88.24)	−41.85%
Female	51.05(30.88,72.51)	27.40(17.14,38.64)	−46.32%
**SDI levels**			
High SDI	6.13(3.81,8.68)	2.11(1.36,2.91)	−65.54%
High-middle SDI	24.28(15.20,34.35)	9.82(6.24,13.49)	−59.57%
Middle SDI	75.92(48.40,109.05)	31.37(19.91,43.16)	−58.68%
Low-middle SDI	216.09(136.99,305.35)	120.73(75.50,166.75)	−44.13%
Low SDI	262.42(164.84,375.30)	150.78(94.72,211.61)	−42.54%
**GDB regions**			
Andean Latin America	74.65(45.20,109.24)	17.27(10.51,25.69)	−76.87%
Australasia	0.85(0.49,1.30)	0.45(0.26,0.68)	−46.67%
Caribbean	26.23(16.71,36.30)	17.67(11.31,25.02)	−32.63%
Central Asia	21.69(13.68,29.58)	24.03(15.32,32.94)	10.80%
Central Europe	10.99(6.86,15.22)	4.62(2.90,6.28)	−57.91%
Central Latin America	43.40(27.73,60.57)	11.23(7.32,15.70)	−74.11%
Central Sub-Saharan Africa	374.96(222.28,557.79)	302.23(181.23,453.00)	−19.40%
East Asia	41.51(25.19,61.14)	6.61(4.00,9.57)	−84.07%
Eastern Europe	10.10(6.35,14.11)	12.03(7.35,17.21)	19.09%
Eastern Sub-Saharan Africa	333.77(208.47,474.65)	165.41(101.49,235.45)	−50.44%
High-income Asia Pacific	13.65(8.40,19.69)	2.64(1.58,3.89)	−80.66%
High-income North America	2.02(1.26,2.85)	0.74(0.47,1.04)	−63.31%
North Africa and Middle East	29.82(17.67,42.65)	13.15(8.28,19.06)	−55.89%
Oceania	149.52(84.73,227.52)	118.45(70.80,177.91)	−20.78%
South Asia	264.77(166.77,375.78)	138.58(87.33,193.60)	−47.66%
Southeast Asia	143.16(88.42,208.96)	66.44(40.56,96.34)	−53.59%
Southern Latin America	11.04(6.74,15.99)	4.76(3.01,6.74)	−56.87%
Southern Sub-Saharan Africa	145.15(91.74,204.10)	160.37(102.19,221.06)	10.48%
Tropical Latin America	23.63(15.01,32.29)	7.92(5.09,10.94)	−66.48%
Western Europe	3.14(1.88,4.66)	0.98(0.61,1.38)	−68.90%
Western Sub-Saharan Africa	138.76(83.53,207.01)	94.18(54.50,143.29)	−32.12%

ASDR, Age-standardized disability-adjusted life years rates; EAPC, Estimated annual percent change; SDI, socio-demographic index

**Fig. 2:**
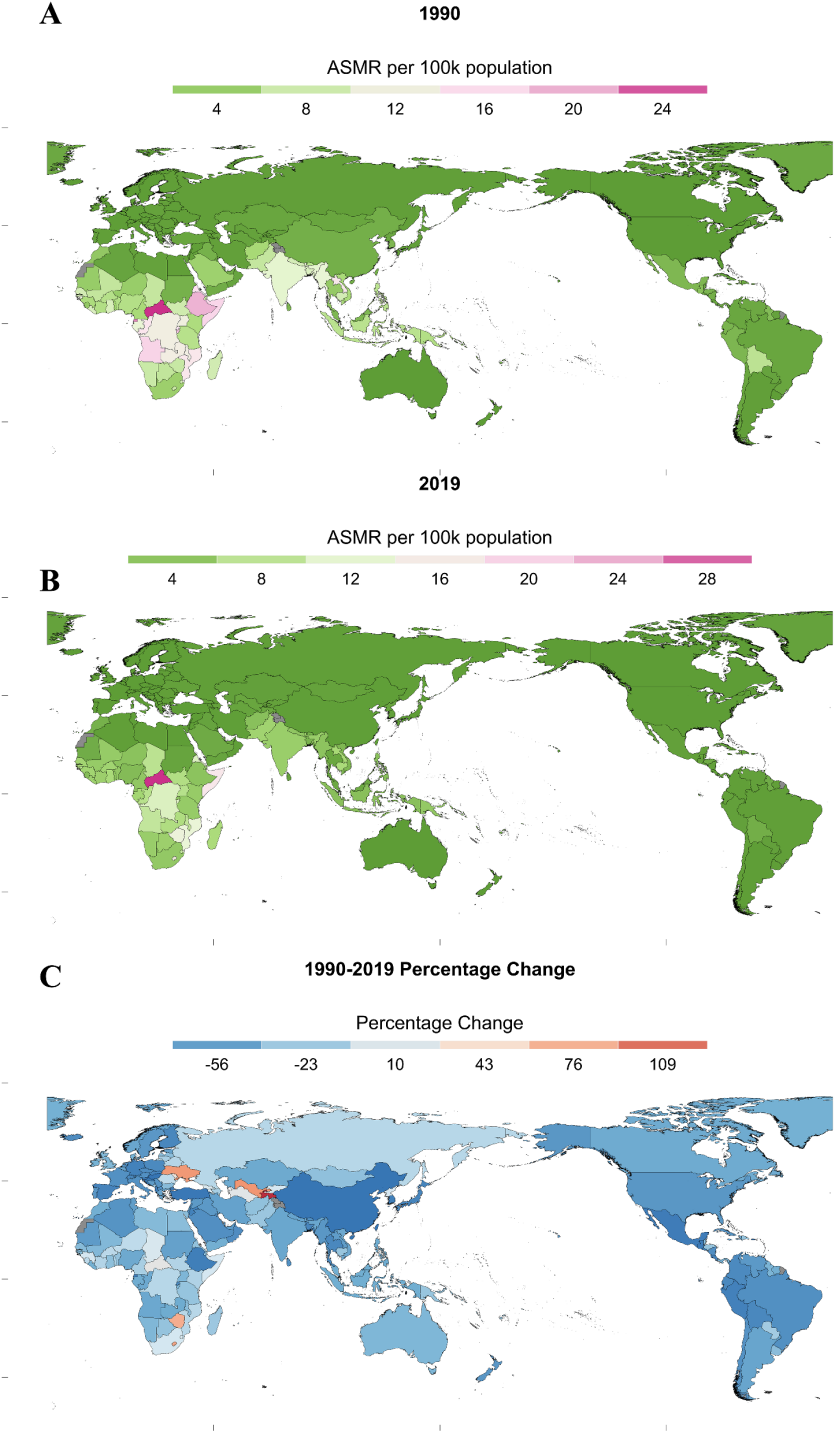
Spatial distributions of age-standardized mortality rates(per 100,000 Population) for tuberculosis attributable to HFPG in 1990(**A**) and 2019(**B**) and 1990–2019(**C**). ASMR, Age-standardized mortality rates; ASDR, Age-standardized disability-adjusted life years rates; HFPG, high fasting plasma glucose

In all 204 countries and territories, the highest TB ASMR and ASDR attributable to HFPG in 1990 occurred in Central African Republic (25.6 and 750.6 per 100,000 populations), followed by Equatorial Guinea (21.9 and 624.0 per 100,000 populations) and Kiribati (20.3 and 641.6 per 100,000 populations), whereas the lowest ASMR and ASDR were observed in Bermuda (0.03 per 100,000 populations) and Australia (0.69 per 100,000 populations) respectively. Thirty years later, in 2019, the highest TB ASMR and ASDR attributable to HFPG still remained in Central African Republic (30.6 and 910.4 per 100,000 populations), followed by Kiribati (19.0 and 582.5 per 100,000 populations) and Somalia (16.9 and 456.1 per 100,000 populations). Meanwhile, the lowest ASMR and ASDR were persisted in Bermuda (0.01 per 100,000 populations) and Australia (0.43 per 100,000 populations) respectively. From 1990 to 2019, Tajikistan experienced the largest percentage increase in the ASMR of HFGP-related TB burden (139.2%) among all 204 countries, followed by Uzbekistan (92.6%) and Ukraine (91.3%), whereas Hungary witnessed the most significant percentage decline (-88.7%). Meanwhile, the country with the largest percentage increment in the ASDR was Ukraine (114.3%), followed by Tajikistan (114.2%) and Lesotho (101.0%), while Singapore experienced the most substantial percentage decrease (−87.9%). (Fig. 2 and Figure S1).

### TB Burden attributable to HFPG by SDI regions

The global TB burden attributable to HFPG by SDI regions was estimated in Tables 1 and 2. Both of the ASMR and ASDR of 5 SDI regions in 1990 was 2–3 times higher than that in 2019. In addition, the ASMR and ASDR of high SDI region, high-middle SDI region and middle SDI region had been at a relatively low level. From 1990 to 2019, high SDI region had the greatest change in ASMR (EAPC: −65.8%) and ASDR (EAPC: −65.54%), while low SDI region had the lightest change in ASMR (EAPC: −43.18%) and ASDR (EAPC: −42.54%).

**Fig. 3:**
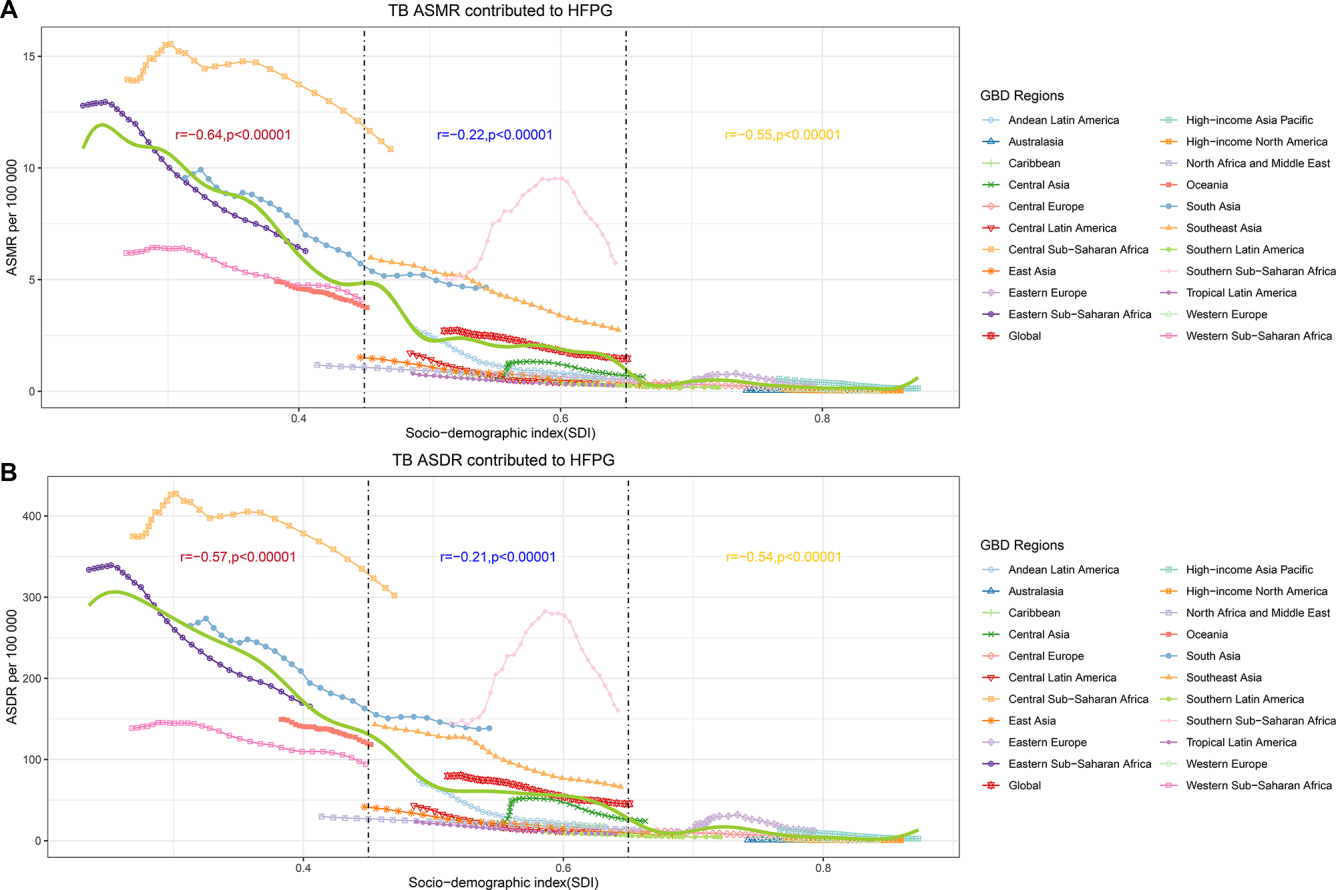
Association between the ASMR(**A**) and ASDR(**B**) of tuberculosis attributable to HFPG and SDI for each country worldwide in 2019. Each dot represents the estimated value of ASDR or ASMR for a specific country or region in a specific year. Pearson correlation coefficients and p-values are shown; SDI, socio-demographic index; ASMR, Age-standardized mortality rates; ASDR, Age-standardized disability-adjusted life years rates; SDI, socio-demographic index; HFPG, high fasting plasma glucose

In order to analyze the relationship between economic development level and TB burden attributed to HFPG, we conducted a correlation analysis in specific SDI regions (Fig. 3). A total of 21 geographic super-regions and global estimation were included in this study. Interestingly, the estimated relationships between SDI and expected ASMR and ASDR for TB deaths attributable to HFPG were both strongly negatively correlated (*r* = −0.64, *p* < 0.00001; *r* = −0.57, *p* < 0.00001) when SDI was less than 0.45. A weak negative correlation was exhibited when SDI was between 0.45 and 0.65 (*r* = −0.22, *p* < 0.00001; *r* = −0.21, *p* < 0.00001), while a moderate negative correlation was observed when SDI was more than 0.65 (*r* = −0.55, *p* < 0.00001; *r* = −0.54, *p* < 0.00001). The ASMR and ASDR for TB attributable to HFPG in Western Sub-Saharan Africa, North Africa and Middle East and East Asia were much higher than expected according to the results of the Gaussian regression. It can be seen that the improvement of economic level may effectively reduce the TB burden attributed to HFPG.

### Burden of TB attributable to HFPG by genders

**Fig. 4:**
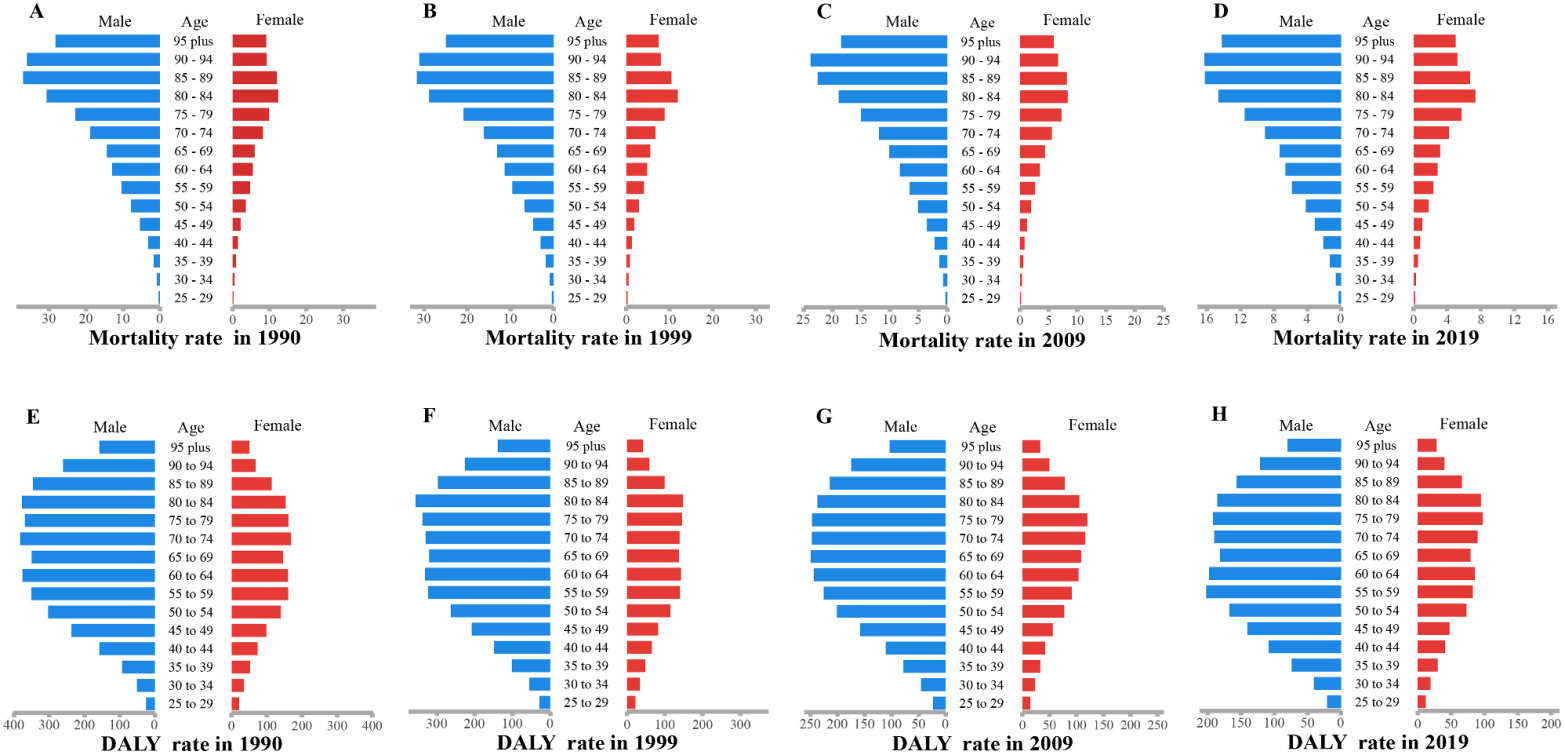
Gender distribution of the mortality and DALY rate for tuberculosis attributable to HFPG for different age groups in 1990(**A**, **E**), 1999(**B**, **F**), 2009(**C**, **G**) and 2019(**D**, **H**). HFPG, high fasting plasma glucose

**Fig. 5:**
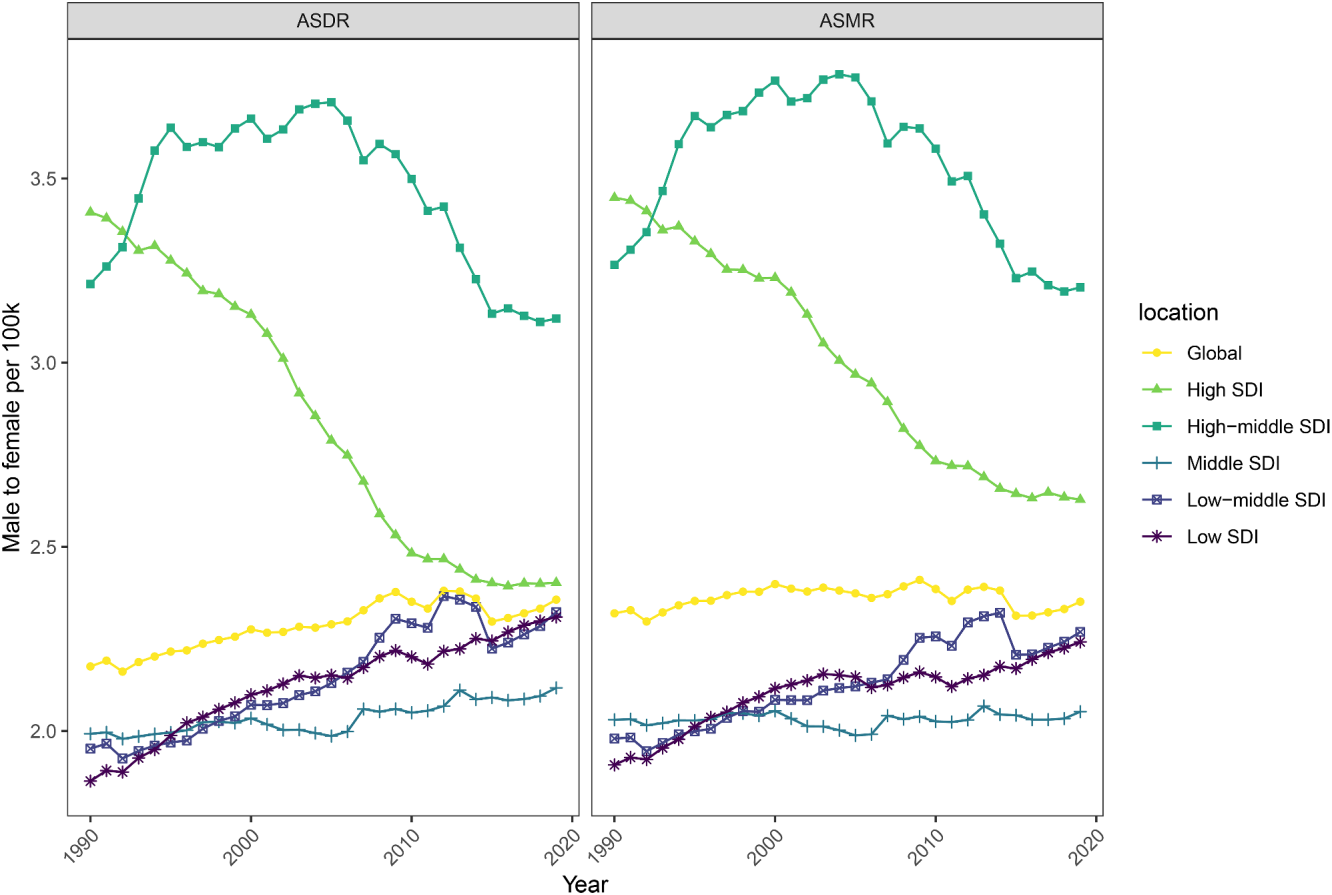
Male to female of ASMR and ASDR for tuberculosis attributable to HFPG in different SDI regions from 1990 to 2019. ASMR, Age-standardized mortality rates; ASDR, Age-standardized disability-adjusted life years rates; SDI, socio-demographic index; HFPG, high fasting plasma glucose

In the analysis of genders, we found that the ASMR and ASDR of TB attributed to HFPG were significantly higher in males than in females across all age groups, and this phenomenon remained robust from 1990 to 2019 (Fig. 4). In addition, the male-female sex ratio of ASMR and ASDR of TB attributable to HFPG in high and high-middle SDI regions showed an overall decreasing trend from 1990 to 2019, especially in high SDI regions, while the ratio in other SDI regions showed a slow increasing trend (Fig. 5).

### Burden of TB attributable to HFPG by different age groups

**Fig. 6:**
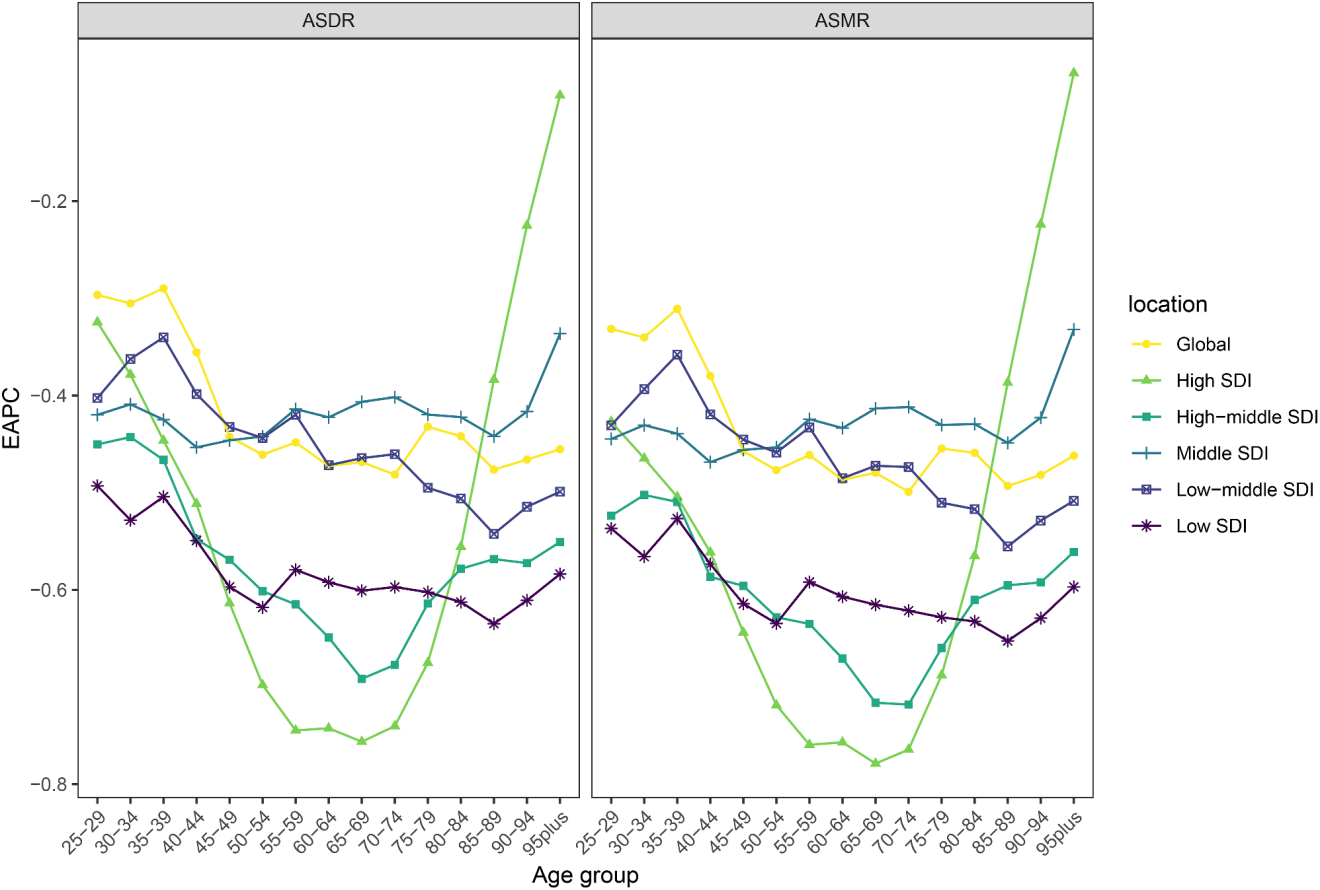
Percentage change of mortality and DALY rate for tuberculosis attributable to HFPG in different SDI regions for all every 5 years age groups. ASMR, Age-standardized mortality rates; ASDR, Age-standardized disability-adjusted life years rates; SDI, socio-demographic index; HFPG, high fasting plasma glucose; EAPC, Estimated annual percent change

Over the 30 years from 1990 to 2019, we found lower declines in both ASMR and ASDR in the middle-aged and the elderly groups (45–79 years old) than in the younger group (< 45 years old) and the older group (> 79 years old) in both high SDI and high-middle SDI regions. Overall, the higher the level of SDI, the greater the decreasing values of ASMR and ASDR by all age group. It should be particularly noted that the decreasing values of ASMR and ASDR for people aged 85 years or older in high SDI regions are lower than all other age groups (Fig. 6).

## Discussion

In this global analysis of 204 countries and territories across 30 years, we assessed global trends and changes in tuberculosis (TB) ASMR and ASDR attributable to high fasting plasma glucose (HFPG) from 1990 to 2019. Our findings show that the TB burden associated with HFPG remained high in 2019, especially in male, older adults, low SDI and low-middle SDI regions. The burden of TB attributable to HFPG has decreased globally, particularly in regions such as East Asia, Andean Latin America and High-income Asia Pacific.

It has been reported that poorly controlled diabetes mellitus (DM) may increase active tuberculosis (TB) risk [14]. On the one hand, HFPG increased the incidence of TB by affecting immune cell function [15]. Elevated glucose concentration can lead to the production of advanced glycation end products and the constant inflammation associated with type 2 diabetes (T2D), ultimately altering the patient’s immune response and facilitating bacterial infection [16, 17]. Due to the change of monocyte in diabetes and opsonins in serum, the ability of monocyte to bind and phagocytosis mycobacterium tuberculosis in patients with poorly controlled glucose is significantly reduced, which will increase the susceptibility to TB [18]. A prospective cohort based on Lu Peng et al. [19] showed that individuals with diabetes increased TBrisk, although it is only observed in diabetes with a BMI less than 24 kg/m^2^. A study from South Korea showed that the course of diabetes is related to the incidence of TB [1]. On the other hand, HFPG can affect the pharmacokinetics of anti-tuberculosis drugs by reducing plasma concentrations, thereby effecting disease treatment [20]. A study conducted by Peru suggested that persistent dysglycemia was associated with unfavorable treatment outcomes in patients with pulmonary TB [21]. In this research, we found that TB disease burden attributed to HFPG across the world remains serious, implying that more attention should be paid to glycemic control.

Firstly, we conduct a comparison of TB burden attributable to HFPG in different SDI regions. SDI is a strong predictor of health-related indicators, emphasizing the significance of income, education, fertility, and cross-sectoral health development actions [22]. Our study revealed that low and low-middle SDI regions experienced a higher TB burden due to HFPG. This could be explained by three factors. Firstly, the proportion of TB patients in developing countries was far higher than that in developed countries, further leading to an increase in the TB burden due to HFPG. The 2022 World Health Organization report on TB pointed out that there were about 10.6 million new cases of tuberculosis in the world in 2021, with more than two-thirds of the global TB burden in 8 developing countries including India, Indonesia, China, Philippines, Pakistan, Nigeria, Bangladesh and the Democratic Republic of the Cong (https://www.who.int/publications/i/item/9789240061729). Secondly, Low-income countries often face challenges with diagnosing and treating TB effectively, especially drug-resistant strains. At present, the success rate of treatment for drug-resistant tuberculosis globally was only 60%, while it has a lower rate in developing countries (https://www.who.int/publications/i/item/9789240061729). Thirdly, low SDI countries also paid less attention to early hyperglycemia or diabetes screening than high SDI countries, for which the lack of funds and health personnel may be the deeper reason [23]. In conclusion, we argue that low SDI countries may represent a weak link in global tuberculosis eradication efforts necessitating additional economic and medical support for improvement.

We further compared the disease burden of TB patients attributed to HFPG by gender, and found that men had a higher disease burden than women across all age groups, which is consistent with many research results [24, 25]. It showed that the incidence rate of male is significantly higher than that of female, with strong evidence that men are disadvantaged in seeking and accessing TB care in many cases [26]. In addition, men tended to have higher rates of tobacco, alcohol use, and other known risk factors for TB [27–30]. Because of men’s role and occupational risks, they are considered to be more likely to be exposed to Mycobacterium tuberculosis [25]. Therefore, it is important to enhance awareness about glycemic control and occupational exposure related to TB among the male population.

After that, we analyzed the change of TB burden in different age groups across 30 years. Although there was a consistent stable trend of percentage change across all age groups, the elderly and middle-aged people in high SDI areas showed higher and lower percentage changes, respectively. We speculated that population aging may be responsible for the low percentage change in high SDI region. Previous studies have shown a higher burden of MTB infection among the elderly [31] and an association between unsuccessful treatment and increasing age [32]. Children and adolescents have reduced resistance to TB infection, coupled with increased opportunities for cross infection in collective life, making them also a high-risk group for TB [33, 34]. From a sociological perspective, middle-aged individuals, being crucial for social development, typically exhibit better health awareness and are more efficient in implementing health policies. Hence, more investment in health resources for projects such as screening, diagnosis, and treatment of TB infection in adolescents and the elderly would be essential in reducing the burden of TB due to HFPG.

To our knowledge, it was the first study to comprehensively analyze the global burden of TB attributed to HFPG, emphasizing the importance of glycemic control strategies aimed at TB control. However, there are some limitations that need to be acknowledged. First, all data used in this study were sourced from GBD 2019, so methodological limitations reported based on the GBD 2019 database also apply here. Second, the TB burden attributed to HFPG globally are estimated values, but inevitably there are measurement errors in this process, which may increase the uncertainty of the estimation results. Thirdly, due to a lack of detailed provincial or state-level data in this study, more specific population reports could not be obtained. In the future, it is crucial not only to protect high-risk populations but also to continue promoting TB control strategies through glycemic control measures.

## Conclusion

Globally, the ASMR and ASDR for TB attributable to HFPG decreased from 1990 to 2019, but remained high in low and low-middle SDI regions, some GBD regions (such as Central Sub-Saharan Africa region, Eastern Sub-Saharan Africa and South Asia), and some high-risk populations (such as the male and the elderly). It is urgent to take effective measures in reducing the high TB burden in these regions and populations. At the same time, it is necessary to strengthen the awareness of early glycemic control and TB treatment.

### Electronic supplementary material

Below is the link to the electronic supplementary material.

Supplementary Material 1

## Data Availability

To download the data used in these analyses, please visit the Global Health Data Exchange at http://ghdx.healthdata.org/gbd-2019.
