# Supplementary material for: Global and regional estimates of tuberculosis burden attributed to high fasting plasma glucose from 1990 to 2019: emphasis on earlier glycemic control
Source: BMC Public Health. 2024 Mar 13;24:782. doi: 10.1186/s12889-024-18260-z (PMC10935816; doi:10.1186/s12889-024-18260-z)
Supplement: Supplementary file 1 — Supplementary Material 1 [file 12889_2024_18260_MOESM1_ESM.docx]

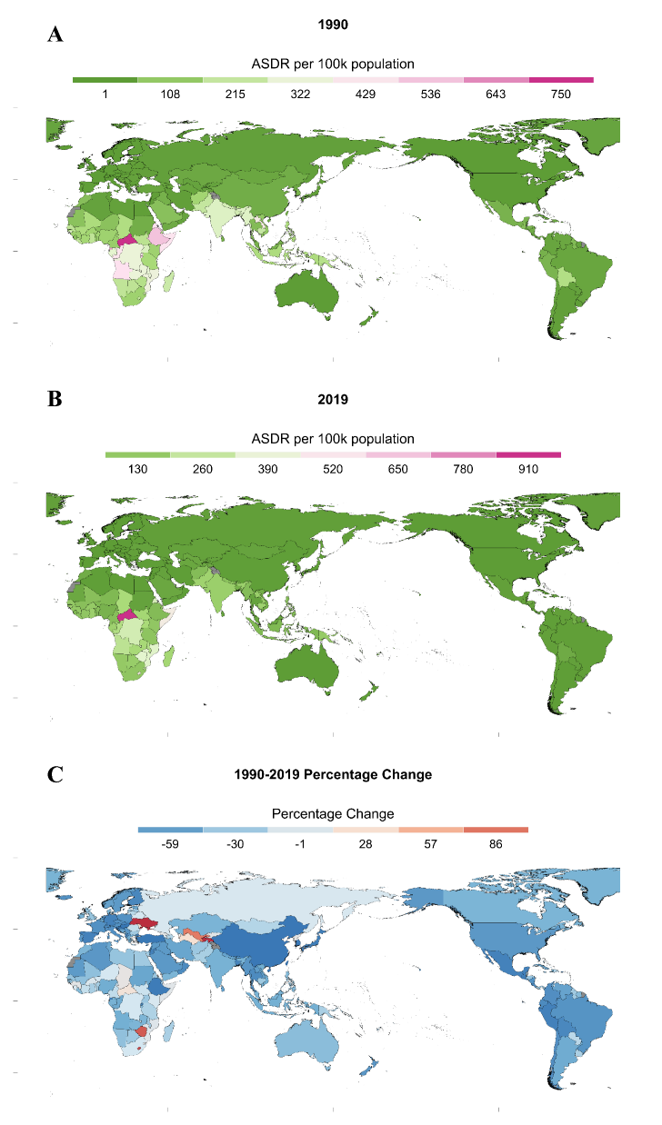


**Figure S1.** Spatial distributions of age-standardized disability-adjusted life rates(per 100,000 Population) for tuberculosis attributable to HFPG in 1990(A) and 2019(B) and 1990-2019(C).
